# Development and Evaluation of Surfactant Nanocapsules for Chemical Enhanced Oil Recovery (EOR) Applications

**DOI:** 10.3390/molecules23071523

**Published:** 2018-06-24

**Authors:** Farid B. Cortés, Mónica Lozano, Oveimar Santamaria, Stefania Betancur Marquez, Karol Zapata, Natalia Ospina, Camilo A. Franco

**Affiliations:** 1 Grupo de Investigación en Fenómenos de Superficie—Michael Polanyi, Departamento de Procesos y Energía, Facultad de Minas, Universidad Nacional de Colombia, Sede Medellín, 050034 Medellín, Colombia; monyka700@gmail.com (M.L.), sbetancurm@unal.edu.co (S.B.M.); kzapataa@unal.edu.co (K.Z.); 2 Grupo de Investigación en Yacimientos de Hidrocarburos, Facultad de Minas, Universidad Nacional de Colombia-Sede Medellín, 050034 Medellín, Colombia; osantamariat@unal.edu.co; 3 Laboratorio de Ciencia de los Alimentos, Departamento de Ciencias, Universidad Nacional de Colombia-Sede Medellín, 050034 Medellín, Colombia; 4 Petroingeniería Regional de Antioquia, Petroraza S.A.S., Calle 80 Sur # 47D 88 Sabaneta, Colombia; nataliaospina@petroraza.com

**Keywords:** adsorption, enhanced oil recovery (EOR), nanocapsules, surfactant, vacuum residue

## Abstract

The primary objective of this study is the synthesis of nanocapsules (NC) that allow the reduction of the adsorption process of surfactant over the porous media in enhanced oil recovery processes. Nanocapsules were synthesized through the nanoprecipitation method by encapsulating commercial surfactants Span 20 and Petro 50, and using type II resins isolated from vacuum residue as a shell. The NC were characterized using dynamic light scattering, transmission electron microscopy, Fourier transform infrared, solvency tests, softening point measurements and entrapment efficiency. The obtained NC showed spherical geometry with sizes of 71 and 120 nm for encapsulated Span 20 (NCS20), and Petro 50 surfactant (NCP50), respectively. Also, the NCS20 is composed of 90% of surfactant and 10% of type II resins, while the NCP50 material is 94% of surfactant and 6% of the shell. Nanofluids of nanocapsules dispersed in deionized water were prepared for evaluating the nanofluid—sandstone interaction from adsorption phenomena using a batch-mode method, contact angle measurements, and FTIR analysis. The results showed that NC adsorption was null at the different conditions of temperatures evaluated of 25, 50, and 70 °C, and stirring velocities up to 10,000 rpm. IFT measurements showed a reduction from 18 to 1.62 and 0.15 mN/m for the nanofluids with 10 mg/L of NCS20, and NCP50 materials, respectively. Displacements tests were conducted using a 20 °API crude oil in a quarter five-spot pattern micromodel and showed an additional oil recovery of 23% in comparison with that of waterflooding, with fewer pore volumes injected than when using a dissolved surfactant.

## 1. Introduction

Worldwide, the global energy demand grows as the world population increases. In fact, it is expected that by 2040 the energy consumption (all energy sources) will increase up to 28% [1]. Regarding petroleum and other liquids, the demand would grow from 95 million up to 113 million barrels per day by 2040 [1]. Further, the oil and gas industry has been continuously looking for new energy sources, as well as for cost-effective alternatives to enhance oil recovery from known reservoirs and thereby cope with the supply of the energy consumption [2]. Currently, primary oil recovery methods roughly enable the production of 5–25% of the original oil in place (OOIP), depending on the nature of the fluids, and the physicochemical characteristics of the porous media [3]. Meanwhile, waterflooding (considered as a secondary recovery method) does provide a recovery of more of 30% of OOIP, and when the reservoir exhibits high horizontal and vertical heterogeneity, this percentage is even lower [3,4]. Hence, alternative processes such as chemical enhanced oil recovery (cEOR) have appeared to increase the productivity of mature reservoirs [2,5]. cEOR methods involve the injection to the reservoir of chemical agents such as alkali, polymer, and surfactants, as well as mixtures between them, with the objective of increasing microscopic and macroscopic sweep efficiency through the viscosification of the injection water and the reduction of interfacial tension (IFT). Mainly, in light and intermediate crude oils, the use of surfactants can increase the capillary number by altering the porous media wettability and decreasing the oil/water IFT, which in turn can reduce the residual oil saturation [6].

The surfactant flooding has been widely studied using experimental and simulation approaches, as well as field trials [5,7,8]. The researchers have focused their efforts on the development of new surfactant structures, selection of the concentration and/or chemical nature for being mixed with co-surfactants looking for ultralow IFT at different reservoir conditions (mineralogy, different quality of saltwater and crude oil, temperature, pressure, among others) and its impact on the oil recovery [9]. However, during the surfactant flooding, the rock surface adsorbs the chemical, limiting its extensive use due to the costs involved [10]. Hence, the reduction of the surfactant adsorption onto rock is a critical parameter in this type of cEOR operations. Further, some researchers have focused their studies on this topic [6,11]. Lorenz et al. [12], French et al. [13], Zhou et al. [14], Dai et al. [15] and Shamsijazeyi et al. [16] employed different chemical agents as additives such as alkali [17,18] and polymers [19,20] to reduce the adsorption phenomena of surfactants by the sacrificial of these agents onto different rocks for surfactant flooding operations. The results obtained in these studies showed the advantages of using these type of sacrificial agents for reducing the adsorption considerably. Nevertheless, this kind of agents can generate strong emulsions, making more complicated the handling of the produced fluids [21,22].

Recently, a few studies have taken advantage of the nanotechnology for improving the surfactant flooding by the inclusion of nanoparticles, mainly of silica gel or surface-modified nanoparticles with chemical agents [23,24,25,26]. It has been observed that under laboratory experiments the recovery of oil increases due to the synergistic effect of nanoparticles and surfactant [27,28]. The incorporation of the nanoparticles into surfactant flooding processes has been assessed through of two methods: (i) nanofluid preparation by the inclusion of nanoparticles in a surfactant-containing solution [29,30] and, (ii) grafting, functionalization or chemical attachment of the surfactant over the nanoparticles surface [27,31]. However, the primary challenge in surfactant flooding is the reduction of the surfactant adsorption [32]. Wu et al. [33] evaluated the influence of the silica nanoparticles on the surfactant adsorption onto the porous media and proposed an inhibition mechanism.

Nevertheless, there are no studies reporting null adsorption of surfactant [25,34], which is an essential parameter for leading the process to cost-effectiveness [32]. Therefore, the primary objective of this study is the development for the first time NC composed of vacuum residue (VR)-isolated type II resins and commercial surfactants, looking for the inhibition of the adsorption phenomena of the chemical agent onto the rock. The core-shell type NC were prepared by the nanoprecipitation method, and these are composed by surfactant as core and VR-isolated type II resins as a shell. The employed surfactants were Span 20 and Petro 50, and the NC were named as NCS20 and NCP50, respectively. The NC were characterized by TEM, FTIR, solubility tests, softening point, entrapment efficiency and DLS. The nanofluids were prepared by dispersing NC in deionized water at different concentrations. The adsorption process of surfactant and/or nanocapsules onto the rock was evaluated using batch-mode adsorption experiments, by UV/Vis spectrometer in the fluid supernatant, and corroborated by contact angle measurements and FTIR analysis on a rock. Additionally, the spinning drop technique was used for determining the minimum achievable IFT, and for selecting the best nanocapsule for carrying out displacement tests in a micromodel. It is expected that the hydrophobic character of the type II resins leads to the strategic liberation of the surfactant in the oil matrix instead of the rock surface, avoiding the waste of surfactant and improving the efficiency of cEOR processes.

## 2. Results

Results are divided into five main sections: (i) nanocapsule (NC) characterization, (ii) nanocapsule adsorption over the rock surface, (iii) IFT measurements, (iv) rheological behavior of NC-containing nanofluids and, (v) enhanced oil recovery tests in a quarter five-spot pattern micromodel.

### 2.1. Nanocapsules Characterization

#### 2.1.1. Size and Morphology of Nanocapsules

**Figure 1:**
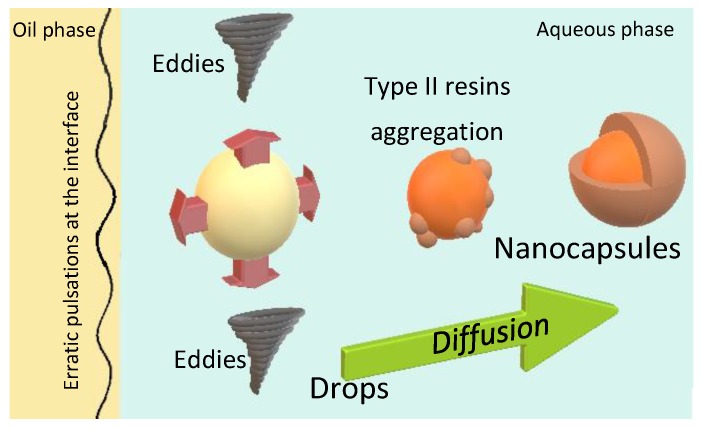
Schematic representation of the interfacial turbulence mechanism for nanocapsule formation based on the nanoprecipitation method also called solvent displacement.

Nanocapsules where synthesized through the nanoprecipitation method also called solvent displacement as described in Section 3.2.2 below. Figure 1 shows a schematic representation of the interfacial turbulence mechanism for nanocapsule formation through the employed method. The nanocapsules are formed by rapid solvent diffusion due to interfacial turbulence by the creation of eddies at the interface that leads to “erratic pulsations” [35]. Further, droplets of nanometric size are formed and stabilized by the stabilizing agent until the aggregation of the type II resins occurs. The nanocapsules are then obtained once the diffusion of the solvent is complete and the growth of the type II resins shell stops.

**Figure 2:**
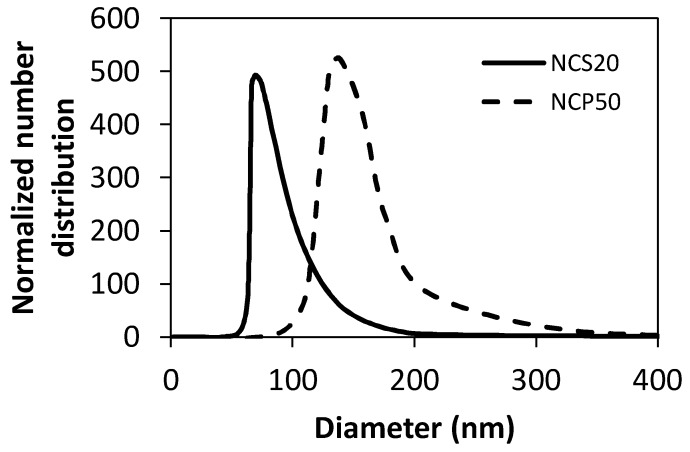
Number particle size distribution of NCS20 and NCP50 samples obtained through dynamic light scattering measurements at 25 °C. Nanocapsules sizes were 71 and 120 nm for NCS20 and NCP50, respectively.

Figure 2 shows the number particle size distribution of the NCS20 and NCP50 samples. The NC mean particle size in the dispersion was determined by dynamic light scattering (DLS) measurements. Results showed that the nanocapsules sizes were 71 ± 4 and 120 ± 6 nm for NCS20 and NCP50, respectively.

It is worth to mention that the mean NC size is independent of the nanocapsules concentration in water, indicating that there is no significant aggregation phenomena between the nanocapsules and hence the stability of the nanofluids is high. This behavior could be due to the resin–resin interaction is low and defined as almost inconsequential [36], contrary to that described by nanoparticles that have a high-density energy surface, which favors the auto-associative phenomena [37].

**Figure 3:**
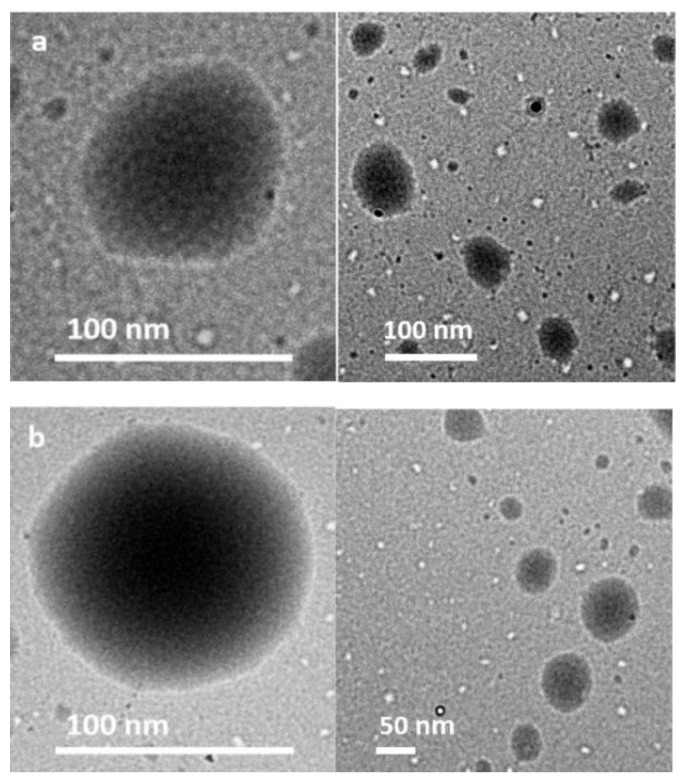
Images of transmission electron microscopy (TEM) at high (**left**) and low (**right**) magnification of (**a**) NCS20 and (**b**) NCP50 samples. The contrast between the core and the shell of the particle confirms the formation of the nanocapsules. NCS20 is composed of 90% of surfactant and 10% of type II resins, and the NCP50 is distributed by 94% of Petro 50 surfactant and 6% of type II resins.

Figure 3 shows the TEM images for (a) NCS20 and (b) NCP50 samples, respectively. The contrast between the core and the shell of the particle confirms the formation of the nanocapsules. The nanocapsules are spherical with uniform shell thickness, and sizes are in agreement with those observed by DLS. It can be observed that the spherical shape could not be rigid due to the liquid content inside the nanocapsule [38]. The results are in agreement with those reported by Guinebretière et al. [38], who synthesized nanocapsules of biodegradable polymers. Also, from the results obtained from TEM, it is possible to conclude that the NCS20 is composed of 90% of surfactant and 10% of type II resins. Similarly, the NCP50 is distributed by 94% of Petro 50 surfactant and 6% of type II resins shell. These results could be related to the higher molecular weight of the Span 20 surfactant that leads to steric hindrance or due to higher hydrophobicity that permits a more efficient physically entrapment (See Span 20 and Petro 50 properties in Section 3.1. below) [39].

#### 2.1.2. FTIR Measurements

**Figure 4:**
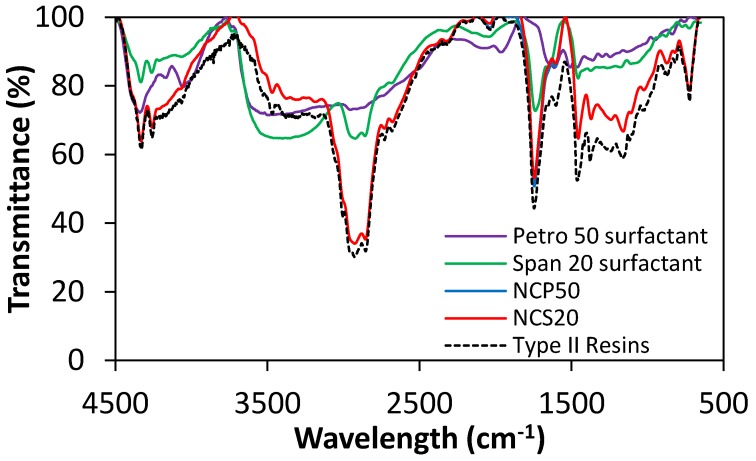
FTIR spectra of vacuum residue-isolated type II resins, Span 20 surfactant, Petro 50 surfactant, and the synthesized NCS20 and NCP50 materials.

FTIR spectrophotometry was used for characterization of Span 20 surfactant, Petro 50 surfactant, type II resins, and synthesized NCS20 and NCP50 materials. The obtained FTIR spectra for each one of the compounds is shown in Figure 4. It can be observed from the FTIR spectrum of type II resins in Figure 4 that main bands associated with aliphatic structures are located at 2852, 2947 and 2922 cm^−1^ for the aliphatic ν C–H for CH_2_ and CH_3_ groups. For δ_as_ C–H of CH_3_ and δ_s_ C–H scissoring of CH_2_, δ_s_ C–H scissoring of CH_3_, ω and τ of the CH_2_ group, and ρ CH_2_ oscillations in phase, the bands are centered at 1455 cm^−1^, 1375 cm^−1^, 1350–1150 cm^−1^, 724 cm^−1^, respectively [40]. Regarding aromatic moieties, the ν C=C of aromatic systems can be observed at 1600 cm^−1^ and C–H, as well as ν C–H of aromatics at 3050 cm^−1^; C–H in condensed aromatic between 1300 and 1100 cm^−1^ and between 1640 and 1600 cm^−1^ [41,42,43,44]. O-related functional groups can be found at 3500–3000 cm^−1^ for ν O–H, and ν C=O at 1744 and 1700 cm^−1^. For S- and N- containing systems, ν S=O of sulfoxide groups are found at 3466 cm^−1^, and bands related to free NH group and C–N single bonds are found at 1375 and 1018 cm^−1^, respectively [41,43,44].

In the case of both Span 20 and Petro 50 surfactants, there is a broadband at 3500–3000 cm^−1^ from O–H groups, C–H in the range 1640–1600 cm^−1^, C–H scissoring of CH_2_ and C–H of CH_3_ at 1450 cm^−1^, C–H scissoring of CH_3_ at 1365 cm^−1^. In the case of Petro 50 surfactant, there is a band related to aromatic C–H bending at 1950 cm^−1^. Also, particularly for the Span 20 surfactant, the C=O of O-containing functional groups can be found at 1732 cm^−1^. The results are consistent with those reported by Khalid et al. [45]. In the case of the nanocapsules, it is observed from Figure 4 that both FTIR spectra of NCS20 and NCP50 are similar to that of type II resins in all the wavelength range due to the transmittance signal is mainly attributed to the shell of the nanocapsules.

#### 2.1.3. Softening Point, Solvency Tests, and Encapsulation Efficiency

Softening point (SP) measurements were conducted following the ASTM E28-14 method [46] to determine the range in which both NCS20 and NCP50 materials work. Results showed that the SP of the synthesized nanocapsules were 113 ± 3 and 115 ± 4 °C for NCS20 and NCP50, respectively, indicating that above of these temperatures, the capsule structure may collapse and the release of the surfactant could occur. Also, it is observed that the obtained SP is independent of the surfactant core and is strongly influenced by the type II resins shell.

Solvency tests of nanocapsules in water and toluene were performed at 25, 50 and 70 °C for different stirring velocities of 100, 1000 and 10,000 rpm. It was observed that there is no solvency of both NCS20 and NCP50 materials in water for all temperatures and stirring velocities evaluated, and is mainly due to the lipophilic character of the resins shell. These results were corroborated by the DLS measurements were nanocapsules with the respective sizes were observed. In contrast, NCS20 and NCP50 materials were completely soluble in toluene under the evaluated conditions. No quantifiable particles were observed after solvency tests in toluene. The encapsulation efficiency was estimated in 74% and 94% in the case of NCS20, and NCP50 materials, respectively, which could explain the higher growth of NCP50 in comparison with NCS20.

### 2.2. Adsorption Experiments

The experimental test of adsorption of the two synthesized NC onto the sandstone were evaluated at different operational conditions of temperature (25, 40 and 70 °C), concentration of the NC (10–10,000 mg/L) and different ratios of the dispersion volume to the dry mass of the sand of 5, 10, and 20 g/L. The adsorbed amount of both NCS20 and NCP50 was evaluated by colorimetry (in the fluid) and TGA (in the rock) and were corroborated through FTIR analyses and contact angle measurements of sandstone before and after adsorption process with the nanocapsules. Both methods (colorimetry and TGA) showed negligible adsorption of NC or surfactant in the sandstone for all the range of concentrations, temperatures, and ratios of dispersion volume to the dry mass of the sand. Results indicate a null affinity between the adsorbent–adsorbate couple, which gives an understanding of the mechanism of NC in enhancing oil recovery processes.

**Figure 5:**
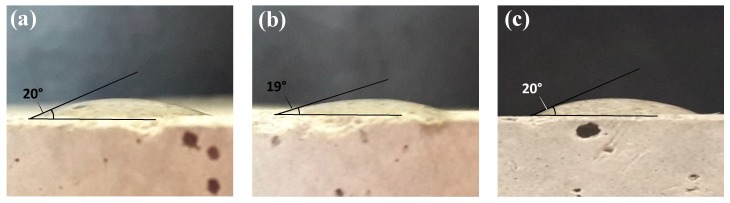
Contact angles for water/air/rock systems (**a**) before, and after contact with dispersions of 10,000 mg/L of (**b**) NCS20, and (**c**) NCP50 materials in deionized water at a fixed temperature of 25 °C.

The interaction between the NC and the sandstone was evaluated through of contact angles in water/air/rock systems before and after of 24 h of contact with the nanocapsules. In all cases, it was observed that the contact angle did not change after contact with both NCS20 and NCP50 materials for all combinations of temperature, NC concentration, and the ratio of NC dispersion volume to dry mass of sandstone. Figure 5 shows the contact angle images for sandstone samples (a) before and after contact with dispersions of 10,000 mg/L of (b) NCS20, and (c) NCP50 materials at 25 °C. As it can be seen in Figure 5, the contact angle for the three samples was estimated in 20 ± 2°, indicating that the water-wet condition of the sandstone is not altered after NC contact and is mainly due to the negligible adsorption of the materials over the rock surface. Variation in contact angles among samples can be explained by differences in surface roughness and heterogeneities.

**Figure 6:**
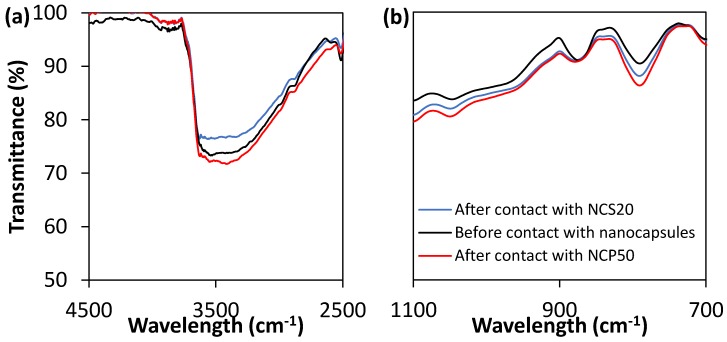
FTIR spectra of sandstone before, and after contact with dispersions of 10,000 mg/L of NCS20, and NCP50 in deionized water, focused on (**a**) O–H region 3500–3000 cm^−1^, and (**b**) fingerprint region for bands centered at 1090, 890, 800, and 690 cm^−1^.

FTIR analysis of sandstone before, and after contact with dispersions of 10,000 mg/L of NCS20, and NCP50 in deionized water was focused in the O–H region at 3500–3000 cm^−1^, as well as in the fingerprint region for bands centered at 1090, 890, 800, and 690 cm^−1^, related to stretching and bending modes of Si–O [47]. Figure 6 shows the FTIR spectra of sandstone before, and after contact with dispersions of 10,000 mg/L of NCS20, and NCP50 in deionized water. It can be observed that there are no significant changes for any of the intensity bands of interest, indicating that the chemical nature of the surfaces is not being altered after contacting the synthesized nanocapsules.

### 2.3. IFT Measurements 

**Figure 7:**
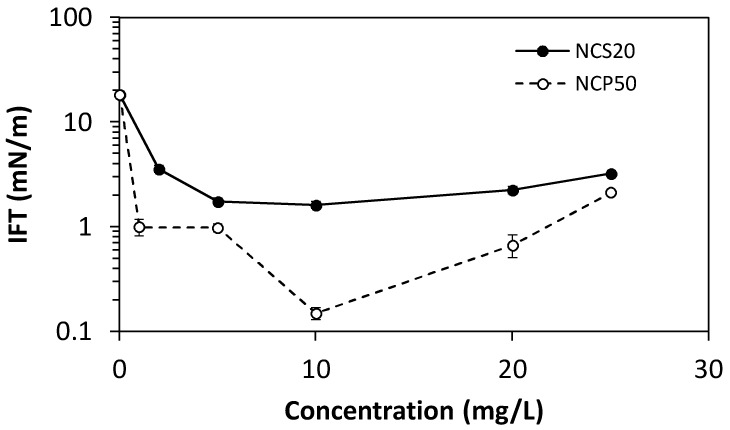
Oil/water interfacial tension in the presence and absence of NCS20 and NCP50 at different concentrations between 1 and 25 mg/L, and at a fixed temperature of 25 °C.

The IFT measurements play a significant role in selecting the best nanocapsule for optimizing the oil recovery. The IFT between crude oil and deionized water was measured at different NC concentrations between 1 and 25 mg/L at a fixed temperature of 25 °C. Figure 7 shows the oil/water interfacial tension in the presence and absence of NCS20 and NCP50 materials. In the absence of NC, the interfacial tension at the oil/water interface was approximately 18 mN/m. The addition of the NC decreased the IFT, and the reduction was higher for NCP50 in comparison with NCS20. The NCP50 showed a reduction in the IFT of two orders of magnitude. The lowest IFT was found at a NCP50 concentration of 10 mg/L, with a value of 0.15 mN/m. For concentrations above 10 mg/L, the values of IFT increase as the concentration of NC dispersed in deionized water increases. This type of behavior was reported previously for other systems [25], which employed surfactants as a chemical agent for the reduction of IFT. This behavior is related to adsorption and desorption of surfactant at the oil/water interface. Surfactant concentration at which the rate of adsorption is equal to the rate of desorption will give the minimum IFT [48]. If the concentration is lower than the one where the minimum achievable IFT is obtained, i.e., at a surfactant concentration lower than 10 mg/L, the rate of adsorption of the surfactant will be higher than the rate of desorption. At 10 mg/L, where the minimum achievable IFT is obtained, these adsorption/desorption rates become equal [48,49]. Further increase in the concentration results in a higher desorption rate from the interface and a higher IFT. It is worth to mention that for cEOR applications an optimum concentration of 10 mg/L is very small suggesting that this type of development as a cost-effective alternative. Further, NCP50 at a concentration of 10 mg/L were selected for displacement tests in a quarter five-spot pattern micromodel.

**Figure 8:**
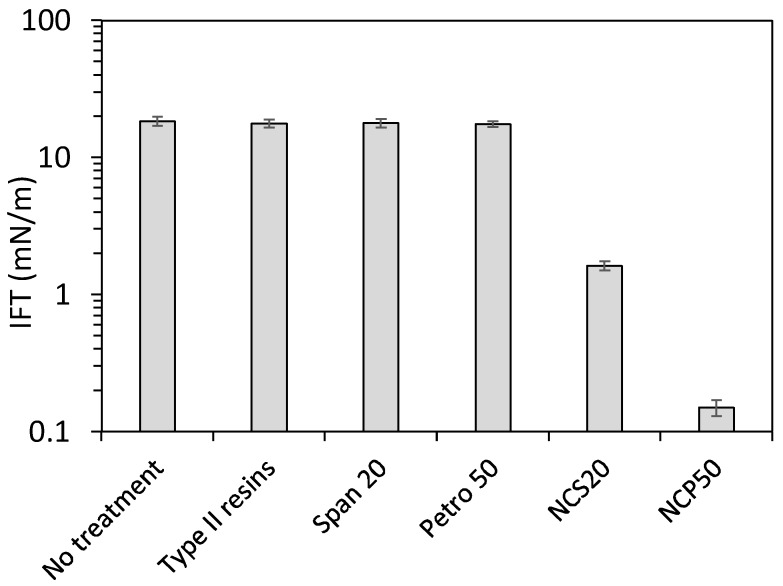
Oil/water interfacial tension in the presence and absence of 10 mg/L of type II resins, Span 20 surfactant, Petro 50 surfactant, NCS20, and NCP50 at a fixed temperature of 25 °C.

Figure 8 shows the individual effect of type I resins and the employed surfactants in the reduction of IFT at 25 °C and at a selected concentration of 10 mg/L. It can be observed that the reduction of IFT when evaluating the individual components of the nanocapsules can be negligible. These results indicate that the hydrophobic character of the type II resins shell would lead to the surfactant positioning at the oil/water interface without losing surfactant in the aqueous matrix. Nevertheless, the effect of nanoparticles needs to be addressed under reservoir temperature and pressure, as one disadvantage could be related to the application under high-temperature conditions due to the thermal stability of the VR shell.

### 2.4. Rheological Behavior of Nanofluid

**Figure 9:**
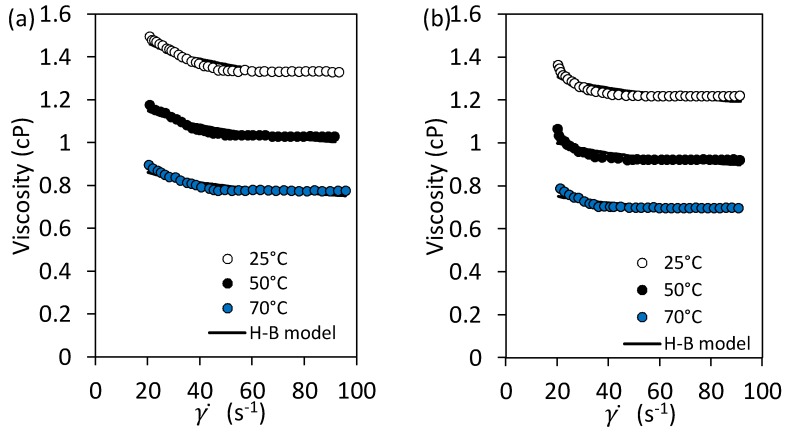
Rheological behavior of nanofluids containing 10 mg/L of (**a**) NCS20 and (**b**) NCP50 materials in deionized water at 25, 50, and 70 °C.

The rheological behavior of the injection fluid in EOR processes is of primary importance to determine the efficiency of the operation regarding injectivity and swept efficiency. According to the IFT measurements, two nanofluids containing 10 mg/L of NCP50 and NCS20 materials in deionized water were prepared and characterized in its rheological behavior at 25, 50, and 70 °C, in a shear rate ($\dot{\gamma }$) range between 1 and 100 s^−1^. Figure 9 shows the rheological behavior of the prepared nanofluids at the three temperatures of evaluation. As expected, the viscosity of nanocapsules slightly decreases as the temperature increases and is explained through the weakening of the interaction forces between the particles [50]. The viscosity is reduced by increasing the system temperature due to the reduction of the attractive binding energy between the molecules, resulting in a decrease in the intermolecular forces between the functional groups in the nanocapsules shell. Also, Figure 9 shows that the viscosity of the NC dispersion is almost constant as the shear rate increases, with a typical behavior of Newtonian fluids [51]. Additionally, results show that for a fixed value of shear rate the viscosity is higher for the NCS20-based nanofluid, in comparison with the one with the NCP50 material.

Table 1 presents the parameters of the Herschel-Bulkley rheological model for the samples evaluated. As observed, the viscosity measured at an infinite shear rate and the consistency index *K_H_* decreased by increasing the temperature. The flow behavior index *n_H_* becomes closer to one, showing a Newtonian behavior. These results are similar to those reported by Taborda et al. [52] who studied the rheology of nanofluids.

### 2.5. Displacement Tests in a Quarter Five-Spot Pattern Micromodel

**Figure 10:**
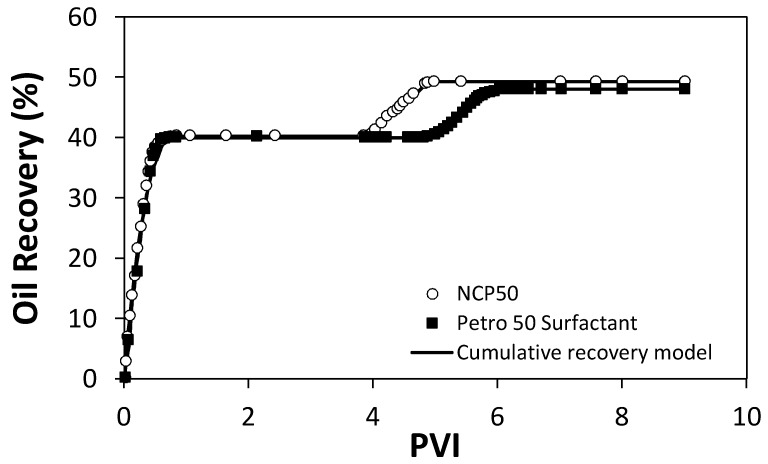
Oil recovery in a quarter five-spot pattern micromodel for the injection of water, followed by aqueous solutions of 10 mg/L of dissolved Petro 50 surfactant, or 10 mg/L of NCP50 material. Tests were conducted at 25 °C and atmospheric pressure.

Displacement tests in a quarter five-spot pattern micromodel were conducted in two steps. First, water was injected up to 4 pore volumes injected (PVI), when no more oil production was observed. Then, 9 mg/L of dissolved Petro 50 surfactant (value obtained according to the encapsulated amount of surfactant in NCP50) or 10 mg/L of dispersed NCP50 material. The primary objective of this section is to compare the oil recovery regarding the *PVI* of the same surfactant concentration, either dissolved or encapsulated. Figure 10 shows the oil recovery behavior for the two displacement tests. Initially, it is observed that in both cases the recovery achieved with water injection is about 48%, indicating the reliability of the experimental setup. For the two displacements tests, surfactant or NCP50 are injected until the production of oil becomes zero, i.e., until the maximum oil recovery is reached. In the case of the nanocapsules, the maximum value of oil recovery is slightly higher than that reached with the dissolved surfactant with values of 49.4, and 48.1%, respectively. Nevertheless, main differences between recovery curves lie in the kinetics of oil recovery for the initial PVI, where it can be observed for the system with NCP50 injection that the additional oil recovery starts immediately and reaches the maximum recovery is after 1.4 PVI. Meanwhile, for the injection of dissolved Petro 50 surfactant, the additional oil recovery begins after 0.8 PVI, and the maximum recovery is reached after 2 PVI.

This delaying effect in the recovery process with dissolved surfactant in comparison with the NCP50 material could be attributed to an adsorption phenomenon of the surfactant over the rock surface, which hinders the positioning of the surfactant at the oil/water interface and delays the expected effect on the recovery of oil. In the case of NCP50, when the material is injected the encapsulated surfactant is released only when it comes into contact with the oil, inhibiting the adsorption over the rock surface to the point that the effect on the recovery is immediate. Table 2 shows the cumulative recovery model parameters for each one of the steps of the displacement tests. From Table 2 it can be observed that the values of *q*_0_ decrease in the order: water injection > NCP50 injection > dissolved Petro 50 surfactant injection. Higher values of *q*_0_ for the water injection are due to the initial oil saturation of the micromodel. Also, higher values of *q*_0_ for NCP50 injection than for dissolved Petro 50 surfactant injection could be related to the inhibition of surfactant adsorption at the rock surface and its immediate effect on the IFT reduction. 

This is corroborated by the *D_i_* values that follow the same trend. *D_i_* is related to the instantaneous changes in the oil recovery. Higher values of *D_i_* indicate that the oil is being recovered faster. As it can be observed, *D_NCP_*_50_ is about two orders of magnitude higher than *D_Petro_*_50_, corroborating that the affinity of nanocapsules with the oil matrix contribute to faster oil recovery.

## 3. Materials and Methods 

### 3.1. Materials

**Figure 11:**
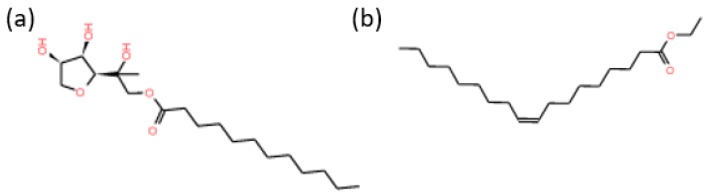
Approximate 2D Structure of (**a**) Span 20 and (**b**) Petro 50 surfactants employed for the preparation of NCS20 and NCP50 materials, respectively.

The type II resins were obtained from petroleum vacuum residue (VR) supplied by a local refinery (Ecopetrol S.A., Barrancabermeja, Colombia). Chromatographic silica (Sigma-Aldrich, St. Louis, MO, USA), *n*-heptane (99%, Sigma-Aldrich), chloroform (99%, Sigma-Aldrich), and methanol (99.9%, Panreac, Barcelona, Spain) were used for type II resins isolation. Diluents such as acetone (99.9%) and toluene (99.8%) were purchased from Sigma-Aldrich and were employed for nanocapsules (NC) preparation. The surfactants used were sorbitan monolaurate (Span 20) and a non-ionic surfactant (Petro 50) supplied by Sigma-Aldrich and Petroraza S.A.S (Sabaneta, Colombia), respectively. Figure 11 shows approximate 2D structures and Table 3 shows some properties of the selected surfactants. Ottawa sand (Minercol S.A., Medellín, Colombia), light crude oil of 20 °API, and KCl (99%, Panreac) were employed for the displacement tests in micromodel. All chemicals were used as received without further purification.

### 3.2. Methods

#### 3.2.1. Isolation of Type II Resins

For the isolation process of type II resins, a VR sample was mixed with *n*-heptane in a ratio of 1:40 g/mL of precipitant [53], sonicated and posteriorly magnetically stirred. Then, the sample was centrifuged for 45 min at 4500 rpm using a Z 306 Hermle Universal Centrifuge (Labnet, Edison, NJ, USA) for separating the maltenes fraction from the asphaltenes [54]. As the petroleum resins can be divided into type I and II resins [55], to isolate the type II resins from the maltenes a particular treatment is required. Initially, maltenes are mixed with the chromatographic silica during 24 h, time enough for reaching the adsorption equilibrium. Then, the obtained sample is filtered, and the supernatant is placed to desorb in a solution of chloroform to methanol at a defined ratio of 4:1 during 24 h. Afterward, the chloroform-methanol mixture is filtered and vaporized, and the type II resins are obtained.

#### 3.2.2. Nanoprecipitation Method for Nanocapsules Preparation

**Figure 12:**
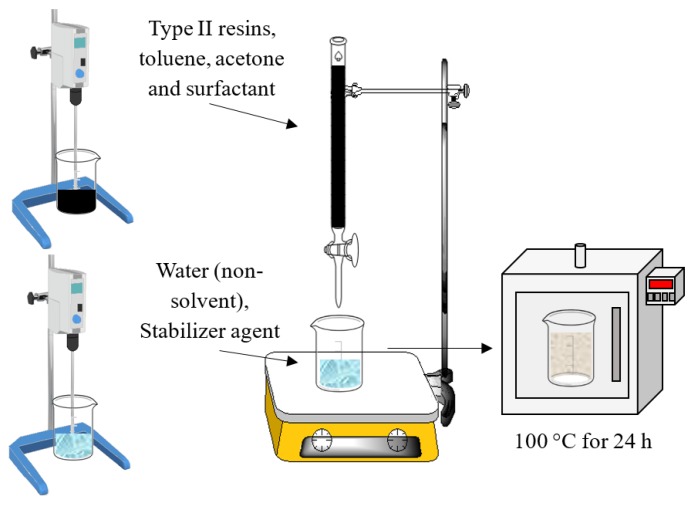
Experimental setup employed for the synthesis of nanocapsules of Span 20 and Petro 50 surfactants in vacuum residue-isolated type II resins.

The nanoprecipitation method is also called solvent displacement or interfacial deposition as described by Fessi et al. [56]. Figure 12 shows the experimental setup employed for the nanocapsules synthesis. The nanocapsule synthesis needs both soluble and non-soluble phases. For this study, the soluble phase consists of a 2:25 mixture of toluene and acetone where the film-forming (shell) type II resins dissolve entirely and encapsulate the commercial surfactant (Span 20 or Petro 50). On the other hand, the non-soluble phase is composed of an aqueous solution containing a hydrophilic surfactant (Tween 20) which acts as a film former. The soluble phase is mixed dropwise with the non-soluble phase with a defined rate of 10 mL per second. Meanwhile, the non-soluble phase remains under continuous stirring at 600 rpm for promoting the formation of the NC. Posteriorly, the temperature is increased to 70 °C to evaporate the acetone from the solution. Finally, the solution is dried at 100 °C to remove the water from the solution. Two NC were synthesized by varying the active chemical compounds in the core.

#### 3.2.3. Nanocapsules Characterization

The NC were characterized using dynamic light scattering (DLS), transmission electron microscopy (TEM), Fourier transform infrared (FTIR), solvency tests, and entrapment efficiency.

For measurement of the NC size, a nanoplus-3 from Micromeritics (ATL, Norcross, GA, USA) equipped with a 0.9 mL glass cell was used, and the mean particle diameter was estimated from the Stokes-Einstein equation [57] for different nanocapsules concentrations between 10 and 1000 mg/L. Morphological analysis was carried out in a Tecnai F20 Super Twin TMP microscope (FEI, Houston, TX, USA). For FTIR analysis, KBr was mixed with each of the obtained nanocapsules and both surfactants in a 30:1 weight ratio [58]. FTIR measurements were conducted using a KCl cell with a 0.25 mm spacing at 25 °C. To estimate the range of temperature in which the nanocapsules work, softening point (SP) measurements were performed following the ASTM E28-14 method [46,54]. Each analysis was performed in triplicate to ensure reproducibility of the measurements.

Finally, the NC was evaluated by solvency tests in polar (deionized water) and non-polar solvents (toluene). The test for the non-polar phase was performed with toluene due to its excellent solvency with type II resins and its aromatic hydrocarbon nature [59]. The solvency test was carried out in insulated vessels using 2.0 mL of solvent at different concentrations of NC between 10 and 10,000 mg/L. The concentration of surfactant was estimated with an RF-6000 spectrofluorophotometer (Shimadzu, Kyoto, Japan) using a calibration curve obtained at a fixed wavelength of 450 nm for the two solvents used. All the tests were performed under different stirring conditions of 100, 1000 and 10,000 rpm at 25, 50 and 70 °C, and atmospheric pressure. DLS measurements were performed after solvency test to verify the absence or presence of nanocapsules in the water and toluene solutions. Also, a measure of the encapsulation efficiency (%EE) of surfactants in the VR-isolated type II resins was determined by TEM and by the mass balance between the added surfactant and the released surfactant in the solvency tests using toluene. The methodology employed to obtain the shell thickness depended on the number of samples and the mapped region. Further, to determine the population size, first the population for each sample is fixed, setting the level of confidence (95%), calculating the confidence interval, and then calculating the population size. The average size of the capsules and the shell thickness was calculated by using a statistical software MiniTab17 (Minitab Inc., State College, PA, USA), and the size obtained for each sample was measured using a morphometric software tpsDig 2.17 [60].

#### 3.2.4. Adsorption Tests

The adsorption tests of nanocapsules were carried out through different approaches in batch–mode experiments by fixing the amount of the sandstone rock sample at 25, 50 and 70 °C according to the procedure described in previous works [61]. The NC were dispersed in deionized water to prepare different dispersions with initial concentrations between 10 and 10,000 mg/L. Then, a fixed mass of sandstone rock sample was added to each one of the prepared dispersions. Different ratios of the dispersion volume to the dry mass of the NC of 5, 10, and 20 g/L were employed. Samples were magnetically stirred at 300 rpm for 24 h. The quantification of NC or surfactant adhered to the rock surface was estimated using different techniques. The amount adsorbed of NC and/or surfactant was determined directly in the rock sample by thermogravimetric analysis (TGA) from 25 °C to 800 °C using a Q50 thermogravimetric analyzer (TA Instruments, Inc., New Castle, DE, USA). TGA experiments were conducted at a fixed heating rate of 5 °C/min under dry air atmosphere and a fixed flow rate of 100 mL/min. Further, colorimetric studies were conducted to evaluate the surfactant release under the evaluated conditions as indirect measurement. For this, a calibration curve of absorbance versus concentration was obtained for each surfactant at a fixed wavelength of 200 nm using a UV-vis spectrophotometer (Genesys 10S, Thermo Scientific, Waltham, MA, USA) [62]. The amount of released surfactant was estimated in the supernatant by absorbance measurements after 24 h of stirring.

Also, wettability tests were carried out to determine the effect of the surfactant on the wettability alteration due to the possible adsorption process. Hence, a first approach for observing the wettability alteration was performed by estimating the contact angle for water/air/rock systems before and after 24 h of contact with the nanocapsules. The water drop onto the surface of the dried sandstone samples was evaluated through image analysis based on the visual estimation of the contact angle for the water/air/rock systems at room temperature in 5 different positions over the rock surface. The droplet volume was injected and controlled with a 5 μL syringe. Further, a photograph of each droplet was taken using a digital camera positioned at a distance of 50 mm from the sample [63]. The obtained images were processed using the LayOut software (Trimble Inc., Sunnyvale, CA, USA), and contact angles were estimated by defining a baseline on the rock surface and fitting the drop profile using sphere- or ellipse-like approximations. Additionally, FTIR analysis was conducted to the rock samples before and after contact with the nanocapsules using an IRAffinity-1S spectrophotometer (Shimadzu, Kyoto, Japan) according to the procedure described in Section 3.2.3 above.

#### 3.2.5. Rheological Behavior of Nanofluid

Rheological measurements of the NC dispersed in water were performed using a Kinexus Pro+ rotational rheometer (Malvern Instruments, Worcestershire, UK) with the concentric cylinders geometry, equipped with a Peltier cylinder cartridge for temperature control. Tests were conducted at 25, 50, and 70 °C in a shear rate range of 1–100 s^−1^ [64].

#### 3.2.6. Interfacial Tension Measurements

The interfacial tension (IFT) experiments were performed for selecting the best concentration of nanoparticles for injection in the displacement tests in micromodels. IFT was estimated at 25 °C using a spinning drop tensiometer SDT (Krüss GmbH, Hamburg, Germany) by adding a drop of oil to the dispersions of nanocapsules at different concentrations between 1 and 25 mg/L. All experiments were repeated three times with standard deviation of approximately 5% with an accuracy of 0.001 mN/m.

#### 3.2.7. Displacement Test in Micromodel

**Figure 13:**
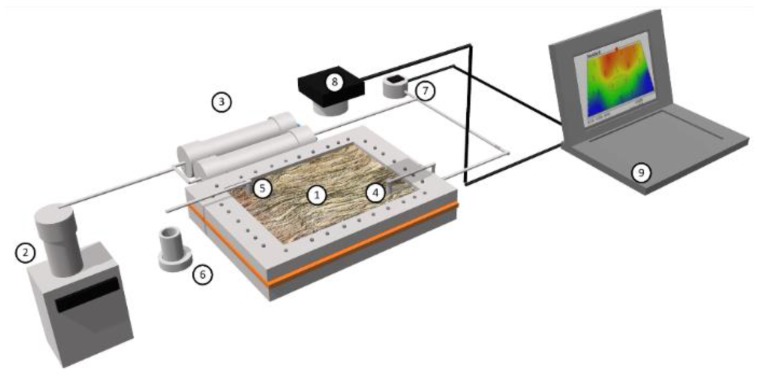
Experimental setup of a quarter five-spot pattern micromodel: (1) Ottawa Sand packing; (2) injection pump; (3) displacement cylinder; (4) injection point; (5) production point; (6) test tube; (7) pressure sensor; (8) digital camera and (9) data acquisition. The dimensions of the cell are 25 cm × 12 cm × 2 cm.

Displacement tests in a micromodel system were performed to evaluate the potential of nanocapsules in enhancing the oil recovery. For this evaluation, three displacement tests were performed in a radial flow cell packed with a 50/60 sieve Ottawa sand. The Ottawa sand was cleaned before experiments as described in previous works [65,66]. The dimensions of the cell are 25 cm × 12 cm × 2 cm. Displacement tests were performed at a fixed temperature of 25 °C and atmospheric pressure. The pore volume (PV) of the system was estimated in 250 mL. The experimental setup for the displacement tests in the radial flow cell is shown in Figure 13 emulating a quarter five-spot pattern of the injector well to the producer well. The configuration is mainly composed by the Ottawa sand packing, an injection pump (Eldex, Napa, CA, USA), stainless steel displacement cylinders, a pressure sensor, a digital camera, and a computer for data acquisition. Nanocapsules were dispersed in deionized water for nanofluid preparation at a determined concentration according to the IFT measurements.

First, the absolute permeability of the system is measured by water injection at a flow rate *q_i_* of 5.0 mL/min. Then, oil is injected at 5.0 mL/min until residual water saturation (Swr) conditions. At this point, oil recovery by water injection is performed, and fluid production is monitored. Water injection stops after determined pore volumes injected (PVI) when oil residual saturation (Sor) conditions are reached. Further, the recovery technology (dissolved surfactant or nanocapsules) is injected, emulating an enhanced oil recovery process. The technology is injected until the recovery of oil is stabilized. Subsequently, water is injected again to ensure that the oil production rate is zero.

### 3.3. Modeling

#### 3.3.1. Herschel-Bulkley Rheological Model

The Herschel-Bulkley (H-B) model was used for describing the rheological behavior of samples as a function of the shear rate $\dot{\gamma }$ (s^−1^) [67,68,69]. The flow behavior index *n_H_* was investigated for different samples and was used as a proxy for the rheological behavior of the fluid. Values of *n_H_* < 1.0 indicate that the fluid follows a pseudoplastic behavior [67]. Conversely, values of *n_H_* ~ 1.0 suggest that the fluid follows a Newtonian behavior. Consistency index *K_H_* (Pa·s^n^) refers to the fluid viscosity. The limiting viscosity parameter *μ_∞__,__γ_* (cP) indicate the behavior of the fluid when subjected to conditions of infinite stresses. The H-B model is described as follows:

(1) $$\mu =K_{{}_{H}}({\dot{\gamma }}^{\left(n_{H}-1\right)})+\mu_{\infty ,\dot{\gamma }}$$

where, *μ* is the viscosity of the fluid at a determined shear rate.

#### 3.3.2. Cumulative Recovery Model

Oil recovery curves were fitted to the cumulative recovery model based on the definition of decline oil rate, as follows [70]:

(2) $$D_{i}=-\frac{1}{q}\frac{dq}{dt}$$

where *q* (mL/min) is the oil rate for determined injection time *t* (dimensionless), and *D_i_* (min^−1^) is the decline rate for determined “*i*” injection step of water, dissolved Petro 50 surfactant, or NCP50 material.

The cumulative oil recovery can be defined as [70]:

(3) $$Np=\int_{0}^{t}q_{0}\cdot e^{-D_{i}\cdot t}dt$$

where *q*_0_ (mL/min) is the oil rate at the beginning of the injection, i.e., when t→0. Further, by integration of the exponential function, the oil recovery at a determined time can be estimated as follows:

(4) $$Np=\frac{q_{0}}{D_{i}}(1-e^{-D_{i}\cdot t})$$

Further, defining the injection time as a function of the PVI as *t* = (*PV·PVI*)/*q_i_*, the cumulative oil recovery can be expressed as:

(5) $$Np=\frac{q_{0}}{D_{i}}\left(1-e^{\frac{-D_{i}\cdot PV}{q_{i}}PVI}\right)=\frac{q_{0}}{D_{i}}\left(1-e^{-50D_{i}\cdot PVI}\right)$$

The accuracy of both H-B model and cumulative recovery model was evaluated by determining the root-mean-square error (*RMSE*%) and the correlation coefficient *R*^2^ [71].

## 4. Conclusions

Nanocapsules of commercial surfactants Span 20 and Petro 50 in a VR-isolated type II resins shell were synthesized by the nanoprecipitation method, with sizes of 71 and 120 nm, respectively. IFT measurements indicated that 10 mg/L of nanocapsules could reduce the IFT up to 99%, showing a synergistic effect between the resins shell and the surfactant core. Also, through softening point measurements, it was determined that the nanocapsules could work at a temperature up to 115 °C. The encapsulation efficiency was estimated in 74% and 94% for NCS20 and NCP50 materials, respectively. The rheological behavior of nanofluids containing the synthesized nanocapsules showed a typical behavior of Newtonian fluids at different temperatures. Also, NCS20-containing nanofluids have a higher viscosity than the one with NCP50 nanocapsules. Through contact angle measurements, it was observed that nanocapsules adsorption over the porous media is negligible, indicating that the surfactant loss can be avoided when the injection is conducted. Displacement tests in a quarter five-spot pattern micromodel show that the nanocapsules can enhance the oil recovery with lower pore volumes injection (43% less) in comparison with the injection of dissolved surfactant.

The oil recovery experiments showed that main differences between recovery curves lie in the kinetics of the initial pore volumes injected. The primary mechanism of nanocapsules EOR is their positioning at the oil/water interface due to its hydrophobic behavior and solvency in non-polar fluids. The release of the encapsulated surfactant only occurs after contacting the oil matrix, which inhibits the adsorption of the active compound over the porous media surface under laboratory conditions. Further studies should include the evaluation of the nanocapsules at reservoir conditions of pressure and temperature. It is expected that this work opens a broader landscape on engineered nanoparticles in nanoparticle/nanofluids enhanced oil recovery (NEOR).

## Figures and Tables

**Table 1:** Parameters of the Herschell-Bulkley rheological model at 25, 50, and 70 °C for the nanofluids containing 10 mg/L of NCS20 and NCP50.

Sample	Temperature (°C)	H-B Model Parameters				
		*K_H_* (Pa∙S^n^)	*n_H_*	*μ_∞,__γ_* (cP)	*R* ^2^	*RSME*%
NCS20	25	3.15	0.9922	1.12	0.96	6.76
	50	2.38	0.945	0.903	0.95	7.64
	70	2.13	0.9722	0.65	0.94	9.12
NCP50	25	2.73	0.9572	1.03	0.87	8.73
	50	2.15	0.964	0.81	0.91	9.73
	70	2.03	0.9812	0.55	0.93	8.76

**Table 2:** Cumulative recovery model parameters for oil recovery in a quarter five-spot pattern micromodel for the injection of water, and aqueous solutions of dissolved 10 mg/L of Petro 50 surfactant, or 10 mg/L of NCP50 material.

Step	Cumulative Recovery Model Parameters			
	*q*_0_ (mL/min)	*D_i_* × 10^−2^ (min^−1^)	*R* ^2^	*RSME*%
Water	3.10	6.83	0.99	1.2
Petro 50 surfactant	0.17	0.14	0.99	3.6
NCP50	0.23	0.05	0.98	7.8

**Table 3:** Properties of type, color, solubility, hydrophilic-lipophilic balance (HLB), and molecular weight (MW) Span 20, and Petro 50 surfactants.

Surfactant	Type	Color	Solubility	HLB	MW (g/mol)
Span 20	Sorbitan monolaurate	Yellow to yellow-green	Water-insoluble	8.6	346.47
Petro 50	Oleic acid ethyl ester	Colorless	More lipid-soluble	10.5	~305
